# Mapping intratumoral coverage of neural stem cell–delivered anti-HER2 antibody in breast cancer brain metastasis

**DOI:** 10.1007/s10585-026-10449-3

**Published:** 2026-11-10

**Authors:** Jawad Fares, Deepak Kanojia, Edgar Petrosyan, Alex L. Tobias, Rafal Chojak, Atique U. Ahmed, Irina V. Balyasnikova, Maciej S. Lesniak

**Affiliations:** 1 Department of Neurological Surgery, Feinberg School of Medicine, Northwestern University, Chicago, IL USA; 2 Malnati Brain Tumor Institute, Robert H. Lurie Comprehensive Cancer Center, Northwestern University, Chicago, IL USA

**Keywords:** Breast cancer brain metastases, HER2, Neural stem cells, Antibody delivery, Tumor coverage, Cell-based therapy

## Abstract

Cell-based therapies can formulate and deliver biologics within the brain, but their development has relied largely on survival outcomes that do not distinguish inadequate payload potency from inadequate tumor distribution. We evaluated intratumoral coverage as a quantitative endpoint for cell-mediated delivery using neural stem cells (NSCs) engineered to secrete an anti-HER2 antibody in breast cancer brain metastasis. Intracranial HER2-positive BT474Br tumors received a single dose of 5 × 10^5^ anti-HER2 antibody-secreting NSCs. Secreted human IgG was reconstructed across consecutive 10-µm sections spanning the entire tumor depth, and therapeutic activity of the same cell dose administered once was assessed in an independent survival experiment. A single NSC dose produced a mean section-level antibody coverage of 60.6%; coverage was ≥ 25% in 96.8% of sections and ≥ 50% in 74.2%. Coverage was greatest in the tumor core and declined toward both poles, a pattern consistent with carrier-cell localization limiting therapeutic reach. The same dose extended median survival from 67 days with vector-control NSCs to 73.5 days (*P* = 0.0009) and increased restricted mean survival by 10.8 days. These data provide an initial spatial benchmark for a therapeutically active dose and establish tumor coverage as a mechanistically informative endpoint for intracranial cell-based delivery.

## Introduction

Brain metastases develop in 30–50% of patients with HER2-positive metastatic breast cancer and remain a principal cause of death in this population [1–4]. Tumor cells that colonize the brain acquire adaptations to the cerebral microenvironment that are associated with therapeutic resistance [5]; consequently, activity against extracranial disease does not ensure equivalent intracranial control. HER2-directed antibodies provide durable systemic benefit but penetrate the intact blood–brain barrier poorly, allowing the central nervous system to become a site of progression despite extracranial response [6–9]. Recent systemic therapies have altered this landscape. Tucatinib combined with trastuzumab and capecitabine produced an intracranial objective response rate of 47.3% and improved overall survival in patients with active brain metastases [10]. Trastuzumab deruxtecan produced intracranial response rates of 73.3% in TUXEDO-1 [11, 12], 71.7% in DEBBRAH [13], and 61.7% in DESTINY-Breast12 [14], reflecting delivery of intact conjugate rather than barrier disruption alone [15, 16].

These advances redefine rather than eliminate the delivery problem. Intracranial responses to active systemic regimens are often partial and finite, the central nervous system remains a frequent site of first progression, and outcomes after failure of both drug classes are poor [4, 17]. Surgery and radiotherapy control macroscopic lesions but do not address infiltrative cells beyond them [18]. In this context, local production of a therapeutic within the brain may complement systemic treatment by sustaining exposure at sites of intracranial disease. Consistent with this rationale, anti-HER2 antibody–secreting neural stem cells (NSCs) combined with tucatinib prolonged survival beyond either agent alone [19].

NSCs exhibit tropism for intracranial pathology and migrate from the implantation site toward tumor mass and microscopic foci beyond the infiltrating edge [20–22]. HB1.F3, a clonal human NSC line, has undergone first-in-human evaluation as a carrier for a prodrug-converting enzyme in recurrent high-grade glioma [23], including administration over multiple treatment cycles without dose-limiting toxicity [24]. The same line carrying the oncolytic adenovirus CRAd-S-pk7 completed a phase I trial in newly diagnosed malignant glioma and advanced to further clinical testing [25]. Locoregional cell therapy has also expanded to CAR T cells [26, 27], mesenchymal stromal cells [28], and intraventricular administration of NSC-delivered virus [29]. Across these platforms and preclinical NSC studies delivering enzymes, cytokines, viruses, and antibodies [30–42], efficacy has nevertheless been assessed predominantly by survival.

Survival is an aggregate endpoint. It establishes therapeutic activity but does not reveal how much tumor was reached, and therefore cannot distinguish an insufficiently potent payload from inadequate distribution. These failure modes require different solutions: one demands a more active therapeutic, whereas the other requires broader delivery. For a payload manufactured in situ, distribution depends on carrier-cell migration and persistence, the distance traveled by the secreted product, and the extent to which overlapping fields cover the tumor. Coverage is therefore an emergent property of the delivery system that must be measured directly.

Direct measurements of coverage are uncommon and have been confined to glioma. A single dose of 5 × 10⁵ oncolytic virus–loaded NSCs covered approximately 31% of patient-derived glioblastoma xenografts [43], whereas a modeling study of NSC distribution in intracranial glioma estimated 75–94% coverage [44]. Quantitative imaging after intraventricular administration [45] and repeated dosing [46] measures carrier-cell localization rather than the tumor fraction exposed to a payload. These estimates may not transfer to brain metastasis, which is generally more circumscribed than glioma and arises within a different stromal and perivascular context. Payload biology also matters: a cell-associated agent reaches tissue entered by the carrier, whereas a secreted antibody may expose adjacent tumor through diffusion.

We therefore evaluated anti-HER2 antibody–secreting NSCs previously shown to produce functional antibody and prolong survival in the BT474Br model after two administrations [47]. The present study instead quantified secreted human IgG after one administration, consistent with the single-dose design used in prior coverage studies. Antibody was reconstructed at 10-µm resolution through the full depth of an established metastasis, and the exposed tumor fraction was quantified. Therapeutic activity of the same one-administration strategy was evaluated in an independent survival experiment.

## Materials and methods

### Cell lines and culture

The brain-tropic, HER2-overexpressing human breast carcinoma line BT474Br was maintained in DMEM supplemented with 10% fetal bovine serum and 1% penicillin–streptomycin. The clonal human neural stem cell line HB1.F3 was maintained under the same conditions. Cultures were held at 37 °C in 5% CO₂, passaged before confluence, confirmed free of Mycoplasma, and authenticated by short tandem repeat profiling. Cells were used within ten passages after thawing.

### Generation of HER2Ab-secreting NSCs

HER2Ab-secreting NSCs were generated as described previously [47]. Briefly, cDNA encoding the heavy and light chains of an anti-HER2 IgG was subcloned into the pLVX-zsGreen1 lentiviral transfer vector. Lentiviral particles were produced by cotransfecting HEK293T cells with the transfer and packaging plasmids, and HB1.F3 NSCs were transduced at low multiplicity of infection. zsGreen1-positive cells were isolated by fluorescence-activated cell sorting to generate a stable polyclonal population. Vector-control NSCs were generated in parallel with empty pLVX-zsGreen1 and sorted identically; the two NSC arms therefore differed only by the antibody transgene. Previous characterization showed that 1 × 10⁶ HER2Ab-NSCs secreted 60, 675, and 962 ng/mL after 24, 36, and 48 h, respectively [47]. Antibody secretion into conditioned medium was confirmed by ELISA before each in vivo experiment in the present study.

### Intracranial tumor implantation and NSC administration

All animal procedures were approved by the Institutional Animal Care and Use Committee and conducted in accordance with NIH guidelines. Female Hsd: athymic nude mice aged 6–8 weeks were housed in a specific-pathogen-free facility. Anesthesia was induced by intraperitoneal injection of 0.1 mL of ketamine HCl (25 mg/mL), xylazine (2.5 mg/mL), and 14.25% ethanol in 0.9% NaCl. After preparation with 70% ethanol, a midline incision was made and a 1-mm parietal burr hole was drilled 2 mm posterior to the coronal suture and 2 mm lateral to the sagittal suture. In a stereotactic frame, 2 × 10⁵ BT474Br cells in 5 µL sterile PBS were injected through a 26-gauge needle to a depth of 3 mm. The needle was withdrawn slowly and the incision was closed with 4 − 0 absorbable suture.

For the coverage experiment, tumors were established for 20 days before a single injection of 5 × 10⁵ HER2Ab-NSCs at the same coordinates. This interval allowed antibody deposition to be measured in a macroscopic lesion rather than a nascent tumor. Animals were euthanized 48 h after NSC administration to capture the deposited antibody field after migration but before substantial carrier-cell attrition. The spatial analysis was designed as a dense, single-lesion reconstruction. Consecutive sections were treated as ordered spatial measurements within one lesion.

For the survival experiment, mice received one intracranial injection through the same burr-hole coordinates seven days after tumor implantation: 5 µL PBS (*n* = 6), 5 × 10⁵ vector-control NSCs (*n* = 8), or 5 × 10⁵ HER2Ab-NSCs (*n* = 8). An investigator not involved in monitoring assigned mice to groups using a computer-generated sequence, and cages were interleaved to limit position effects. Investigators assessing morbidity endpoints were blinded to treatment assignment. Group sizes were based on our previous study in this model [47]; no formal power calculation or interim analysis was performed. Animals were monitored at least three times weekly for 120 days and euthanized upon reaching predefined IACUC morbidity criteria. Euthanasia was recorded as the survival event. No animals were censored or excluded.

### Histology and immunohistochemistry

Brains were harvested, fixed in 4% paraformaldehyde for 72–96 h, paraffin-embedded, and sectioned serially in the coronal plane at 10 μm through the entire lesion. Hematoxylin and eosin staining was performed on every tenth section to delineate tumor boundaries. Secreted human antibody was detected on serial sections with an HRP-conjugated anti-human IgG and visualized with 3,3′-diaminobenzidine (DAB) and hematoxylin counterstain. Because the host was athymic and the payload was human IgG, intralesional DAB signal represented NSC-secreted antibody. Whole-section images were acquired at ×10 with a Nikon Eclipse E200U and at high resolution with an Automatic Cellular Imaging System (ChromaVision Medical Systems). Sections were ordered from anterior to posterior, with depth defined as cumulative distance along this axis. The anterior and posterior poles corresponded to the first and last tumor-containing sections, respectively. The injection tract was not independently labeled and therefore could not be localized precisely within the reconstructed volume.

### Quantification of tumor coverage

Coverage was quantified section by section with ImmunoRatio, an ImageJ plugin that automatically separates DAB-positive and hematoxylin-only areas using its built-in adaptive thresholding and reports the DAB-stained fraction of total nuclear area [48]. The same automated workflow was applied to every section. For each consecutive evaluable section spanning the entire tumor depth, the tumor region was outlined on the corresponding H&E section and transferred to the DAB image. The ImmunoRatio labeling index within the region was recorded as percent tumor coverage at that depth. An investigator blinded to depth analyzed sections in randomized order. Staining intensity was not quantified; coverage therefore represents histologically detectable antibody deposition rather than antibody concentration or functional exposure. Each antibody-secreting NSC was modeled with an effective radius of action of 100 μm, consistent with previous estimates of secreted-protein diffusion from transplanted carrier cells in brain parenchyma [44, 47, 49]. This radius defined the area of effect used to relate discrete cell positions to the continuous coverage field.

### Statistical analysis

Survival was estimated by the Kaplan–Meier method and compared across the three groups with the log-rank test. Pairwise log-rank comparisons were adjusted by the Holm method, and a log-rank test for trend evaluated the ordered groups (vehicle < vector-control NSC < HER2Ab-NSC). Cox proportional-hazards estimates were unstable because event times were clustered and heavily tied; hazard ratios were therefore not reported. Treatment effect was instead expressed as the difference in restricted mean survival time through day 96, the longest observed event time. This estimator uses the full survival curve and is not destabilized by tied events. Confidence intervals for median and restricted mean survival were obtained by resampling.

Tumor coverage was summarized descriptively across consecutive sections and analyzed as a depth-ordered spatial series. Because all sections originated from one lesion, they were treated as spatial subsamples rather than independent biological replicates, and no population-level confidence intervals or regional hypothesis tests were calculated. Within-lesion spatial organization was characterized using the lag-1 autocorrelation coefficient and comparison with a null distribution generated by random ordering along the depth axis. The full autocorrelation function was examined to 150 μm to estimate the distance over which coverage remained correlated. Regional patterns were described after dividing the depth axis into equal anterior, middle, and posterior thirds. Beyond the coverage maximum, the relationship between coverage and depth was summarized using Spearman correlation and the slope of a least-squares linear fit. A segmented least-squares model identified depths at which the coverage gradient changed. Analyses were performed in Python 3.13 using lifelines, SciPy, and NumPy. Individual-animal survival times and section-level coverage values are provided as source data.

## Results

### Antibody-secreting NSCs as a distributed intratumoral source

**Fig. 1:**
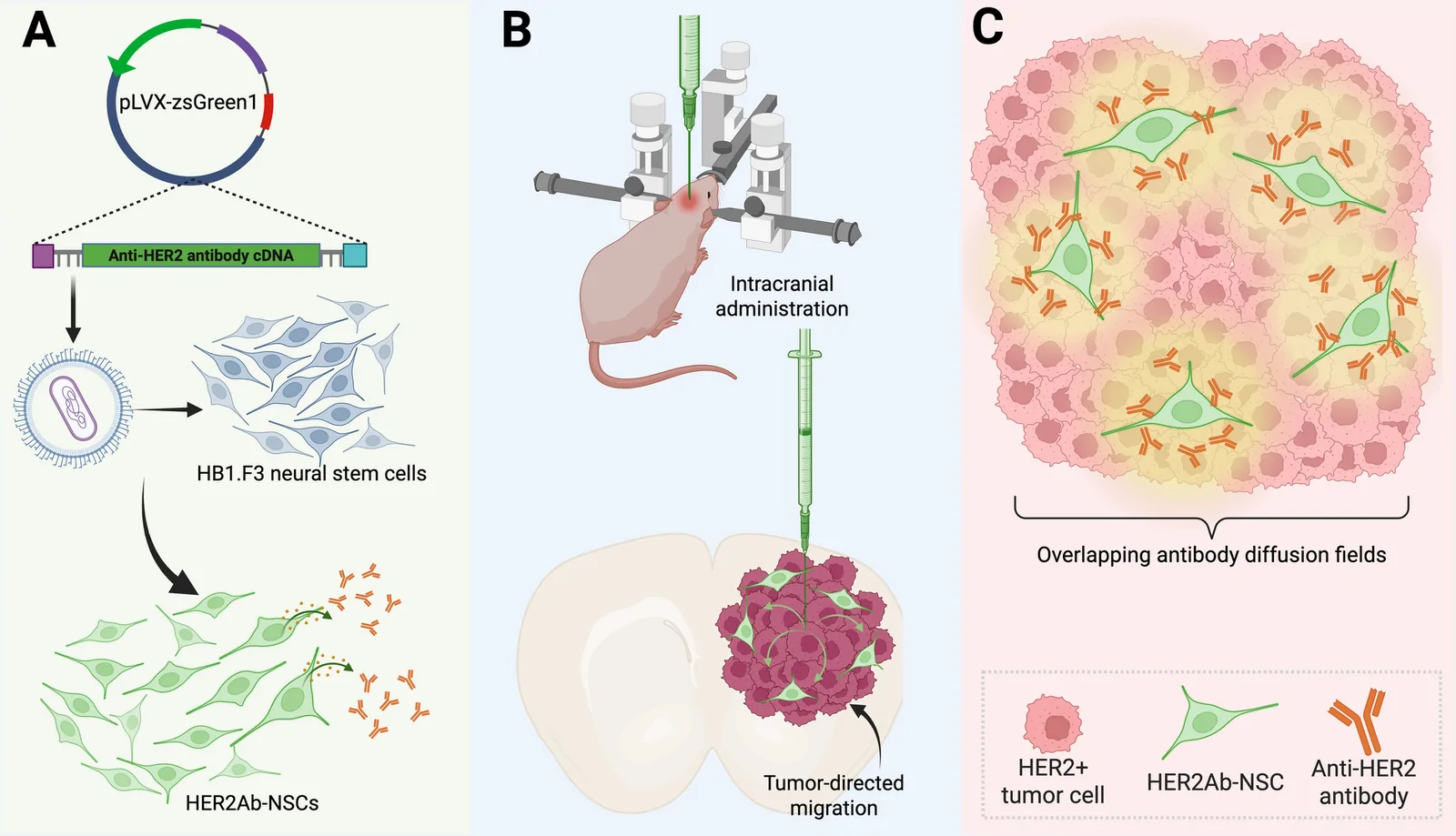
Neural stem cells as a distributed intratumoral source of anti-HER2 antibody. **A** HB1.F3 neural stem cells were transduced with pLVX-zsGreen1 encoding anti-HER2 antibody to generate HER2Ab-NSCs. **B** Following intracranial administration, HER2Ab-NSCs migrate through the metastasis. **C** Secreted antibody forms overlapping diffusion fields throughout the tumor

HB1.F3 human NSCs were transduced with a lentiviral vector encoding an anti-HER2 IgG, yielding a stable population that secretes functional antibody capable of binding HER2-overexpressing breast cancer cells, inhibiting proliferation, and suppressing downstream HER2 signaling [47, 49] (Fig. 1A). After intracranial administration, the transduced NSCs migrate from the injection site into the surrounding lesion (Fig. 1B). Antibody secreted along these trajectories generates overlapping diffusion fields within the tumor (Fig. 1C). This design predicts distributed exposure but does not establish what fraction of tumor is reached.

### A single dose of HER2Ab-NSCs prolongs survival relative to cell-matched controls

**Fig. 2:**
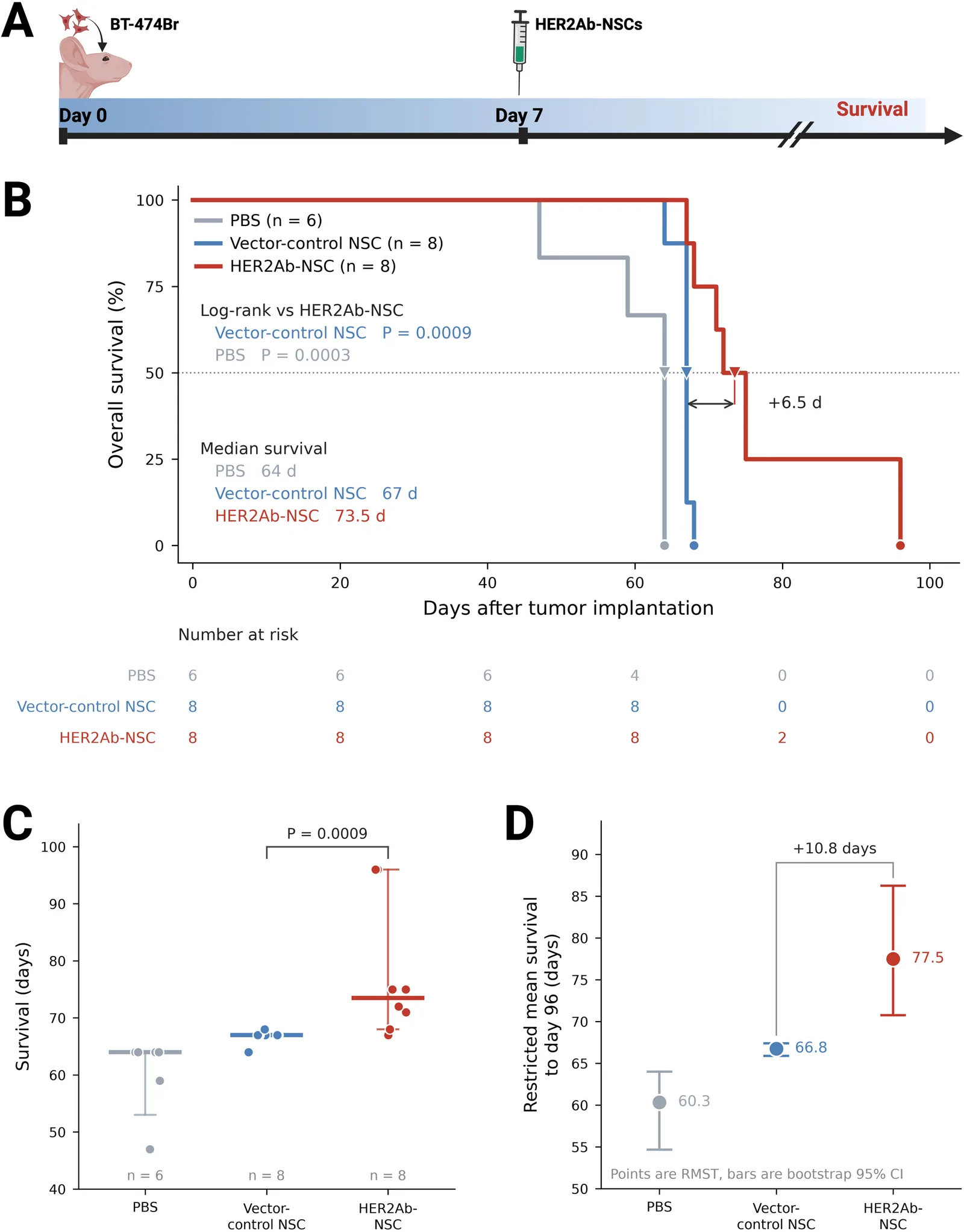
A single dose of HER2Ab-NSCs prolongs survival. **A** Mice bearing intracranial BT474Br xenografts received vehicle (*n* = 6), vector-control NSCs (*n* = 8), or HER2Ab-NSCs (*n* = 8) seven days after tumor implantation and were followed to endpoint. **B** Kaplan–Meier curves with numbers at risk. Triangles indicate median survival (64.0, 67.0, and 73.5 days); overall log-rank *P* = 1.6 × 10⁻⁶. Pairwise P values compare HER2Ab-NSCs with each control. **C** Individual survival times with medians and 95% confidence intervals. **D** Restricted mean survival to day 96 with 95% confidence intervals; HER2Ab-NSCs increased survival by 10.8 days versus vector-control NSCs. No animals were censored or excluded

Previous work with the same BT474Br model and HER2Ab-NSC line used two administrations, one and two weeks after tumor implantation, and extended median survival from 66.0 to 95.5 days [47]. Because the present study was designed to quantify coverage after one administration, we conducted a new survival experiment using only the first of the two previously established treatment time points. This experiment was intended to establish whether one administration at the cell dose used for coverage remained therapeutically active, rather than to reproduce the larger effect of the prior two-dose regimen. Mice bearing intracranial BT474Br xenografts received one injection of vehicle (*n* = 6), vector-control NSCs (*n* = 8), or HER2Ab-NSCs (*n* = 8) seven days after tumor implantation and were followed for 120 days (Fig. 2A). All 22 animals reached a survival endpoint; none were censored or excluded.

Survival differed across the three groups (Fig. 2B; log-rank *P* = 1.6 × 10 − 6; test for trend *P* = 6.2 × 10 − 7). Median survival was 64.0 days with vehicle, 67.0 days with vector-control NSCs, and 73.5 days with HER2Ab-NSCs. Both comparisons involving HER2Ab-NSCs remained significant after Holm adjustment: versus vehicle, *P* = 0.0003 (adjusted *P* = 0.0009); versus vector-control NSCs, *P* = 0.0009 (adjusted *P* = 0.0018).

Individual event times further distinguished the groups (Fig. 2C). Seven of eight vector-control animals died between days 64 and 67, whereas survival in the HER2Ab-NSC group ranged from 67 to 96 days and included two animals surviving to day 96. Restricted mean survival through day 96 was 60.3 days with vehicle, 66.8 days with vector-control NSCs, and 77.5 days with HER2Ab-NSCs. The HER2Ab-NSC group gained 10.8 days relative to the cell-matched control (Fig. 2D). Thus, a single dose shifted the survival distribution and produced a longer-surviving tail absent from both control groups.

### Secreted antibody is detectable throughout an established metastasis

**Fig. 3:**
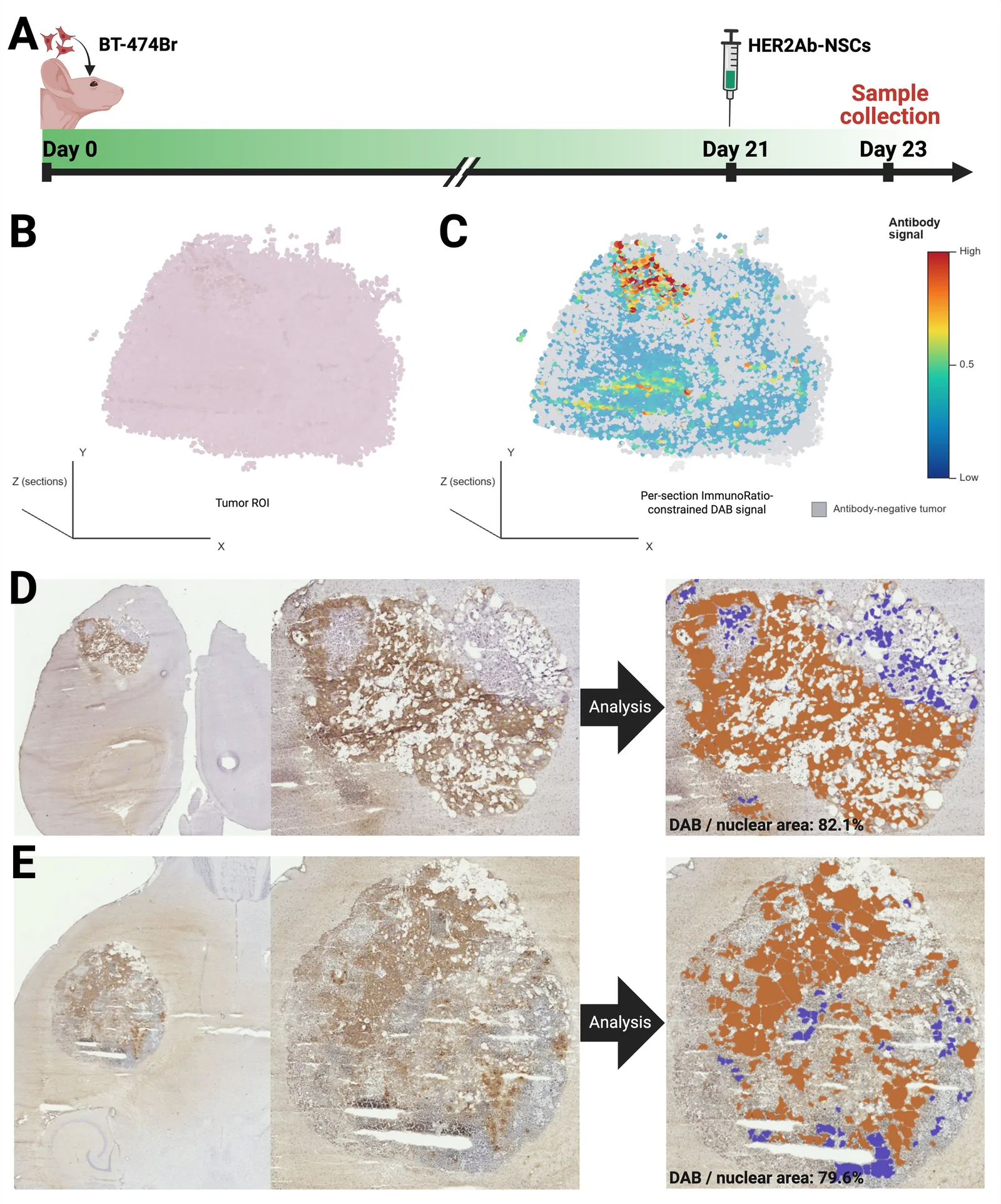
Detection and segmentation of secreted human IgG within an established metastasis. **A** BT474Br metastases were established for 20 days, treated with 5 × 10⁵ HER2Ab-NSCs, and harvested 48 h later. **B** Tumor regions across serial sections were reconstructed volumetrically; X and Y denote within-section coordinates and Z denotes depth. **C** Per-section antibody signal mapped onto the tumor volume. Grey indicates no detectable antibody. **D, E** Representative sections showing the tumor at low and high magnification and after automated segmentation. Antibody-positive nuclear area is reported as percentage coverage. Human IgG signal identifies detectable NSC-secreted antibody throughout the tumor-bearing parenchyma

We next mapped the antibody field generated by one dose. BT474Br metastases were established for 20 days before one intracranial administration of 5 × 10⁵ HER2Ab-NSCs (Fig. 3A), allowing deposition to be evaluated in a macroscopic lesion. Brains were harvested 48 h later to capture the field after NSC migration but before substantial carrier-cell attrition. In the athymic host, immunodetection of human IgG within the lesion specifically reported the NSC-secreted payload.

Consecutive 10-µm sections spanning the entire tumor depth were evaluated. Tumor regions were outlined on every section and reconstructed as a volume (Fig. 3B). Mapping section-level antibody signal onto this volume showed exposed and unexposed regions within the same lesion (Fig. 3C). Human IgG was distributed through tumor-bearing parenchyma. Automated segmentation distinguished DAB-positive from unlabeled nuclear area and returned percent coverage at each depth (Fig. 3D and E).

### Coverage is spatially organized and highest in the tumor core

**Fig. 4:**
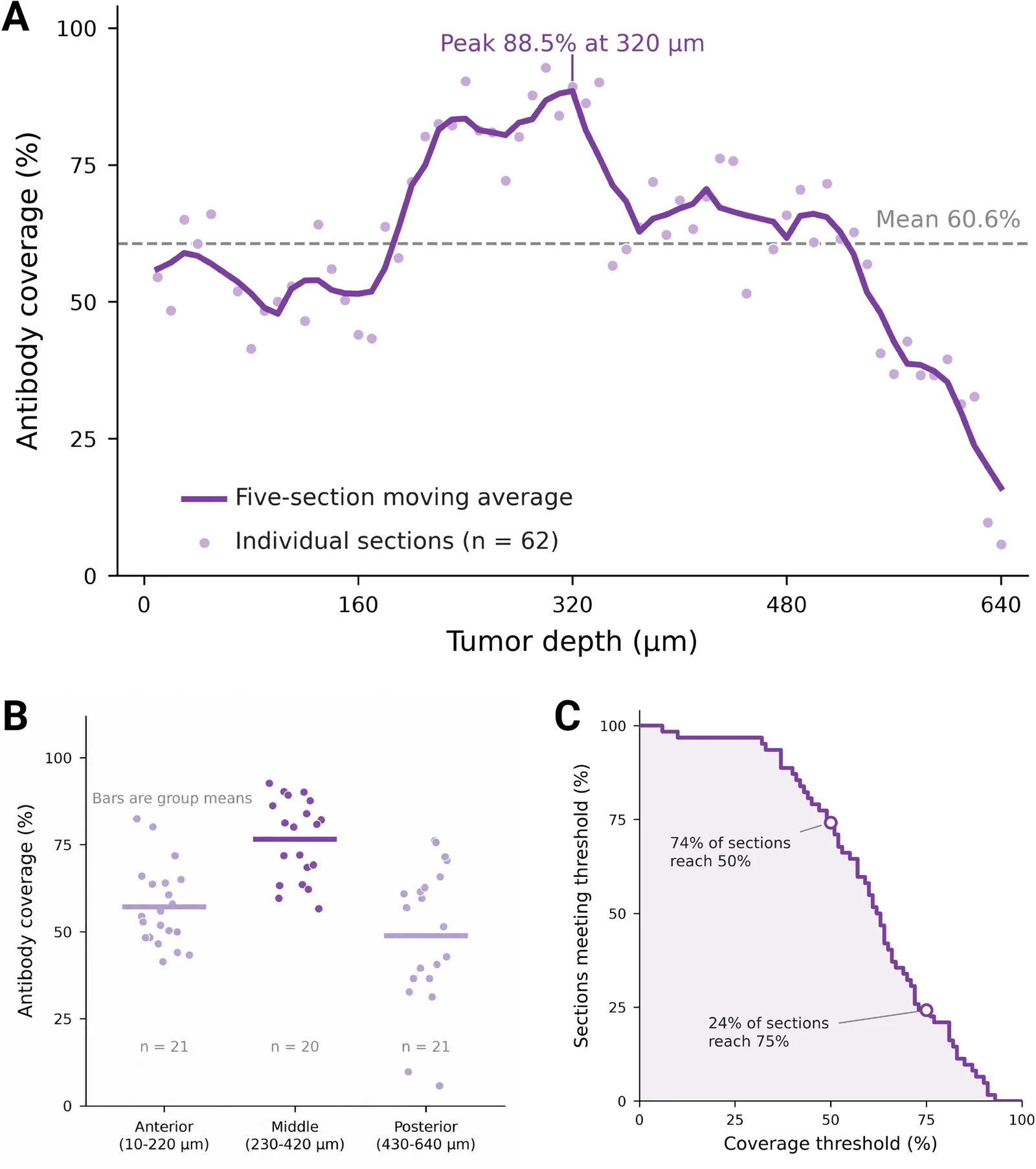
Antibody coverage is substantial, graded, and spatially organized. Consecutive 10-µm sections spanning the lesion were quantified as in Fig. 3. **A** Coverage by depth with a five-section moving average and the lesion-wide mean of 60.6%. Coverage peaked at 88.5% near 320 μm and declined beyond the peak by 17.5% per 100 μm (Spearman ρ = −0.79). **B** Descriptive distribution of coverage across equal anterior, middle, and posterior thirds. Bars indicate means; points represent consecutive sections from the same lesion. **C** Proportion of sections reaching each coverage threshold; 74% had ≥ 50% coverage and 24% had ≥ 75% coverage

Across the reconstructed lesion, mean section-level coverage was 60.6%, ranging from 5.7% to 92.7% (SD, 18.8%; coefficient of variation, 31%) (Fig. 4A). Coverage did not vary monotonically along the anterior-to-posterior depth axis. The five-section moving average reached 88.5% near 320 μm and decreased in both directions. Mean coverage was 57.1% (SD, 11.6%) in the anterior third, 76.6% (SD, 11.6%) in the middle third, and 48.8% (SD, 20.0%) in the posterior third (Fig. 4B). A segmented linear model identified changepoints at 163 μm and 243 μm, bracketing a steep increase toward the core (R² = 0.74). Beyond the maximum, coverage declined by 17.5% points per 100 μm (Spearman ρ = −0.79) and reached 9.7% and 5.7% at 630 and 640 μm, respectively.

Low-level exposure extended through nearly the entire lesion, whereas higher coverage was less frequent. Coverage was ≥ 25% in 96.8% of sections, ≥ 50% in 74.2%, ≥ 75% in 24.2%, and ≥ 90% in 4.8% (Fig. 4C). Thus, antibody was detectable across nearly all depths but covered most tumor area in only a subset of sections.

The nearly 20-fold variation across a lesion less than 1 mm deep could reflect measurement noise or spatial organization. Adjacent 10-µm sections were strongly correlated (lag-1 *r* = 0.855, *P* < 10⁻⁴ against the randomly ordered null), and correlation declined smoothly with separation. It remained above the 95% null bound to approximately 100 μm before approaching zero. Antibody deposition therefore formed coherent domains approximately 100 μm across rather than varying independently between sections. This scale corresponds to the effective radius of antibody action used in the analysis and to previous estimates of secreted-protein diffusion from transplanted carrier cells in brain parenchyma [43, 44, 49].

## Discussion

Cell-based delivery platforms have advanced to clinical testing in brain tumors largely on the basis of survival outcomes [23–27]. Survival demonstrates activity but does not establish whether a payload reached the intended tumor volume. We quantified this delivery dimension through dense spatial reconstruction of one intracranial BT474Br xenograft. Within the reconstructed lesion, one dose of antibody-secreting NSCs produced a mean section-level coverage of 60.6%; coverage was ≥ 25% in 96.8% of sections and ≥ 50% in 74.2%. A separate survival experiment demonstrated that a single administration of the same cell dose was therapeutically active. Its more modest survival benefit relative to the previously reported two-dose regimen is consistent with the lower treatment intensity and should be interpreted in the context of the study design: the survival experiment was included to establish the activity of a single administration, not to optimize efficacy.

The measured coverage also provides context for other cell-based platforms. Oncolytic virus–loaded NSCs at the same cell dose covered approximately 31% of patient-derived glioblastoma xenografts [43]. Although the coverage observed in the reconstructed lesion was nearly twice as high, the studies differ in tumor model, payload, and analytical approach, precluding attribution to any single factor. A secreted antibody can expose tissue beyond the position of its carrier cell, whereas a cell-associated payload remains spatially coupled to the carrier. Tumor geometry may also contribute because a circumscribed metastasis presents a different delivery problem from infiltrative glioma. Coverage estimates should therefore not be transferred across payloads or tumor types without direct measurement. Moreover, no validated coverage threshold defines therapeutically sufficient exposure for a cell-delivered antibody, so the observed 60.6% cannot yet be classified as adequate or inadequate. The apparently uncovered fraction represents a potential delivery limitation, but its biological significance depends on antibody concentration, exposure duration, HER2 density, receptor occupancy, and downstream effects not measured here.

The clinical rationale for intracranial delivery has also changed. Tucatinib-based therapy and trastuzumab deruxtecan produce intracranial responses in many patients [10, 11, 14], so cell-based delivery can no longer be justified solely by an inability of antibodies to enter the brain. Nevertheless, responses are often partial, the central nervous system remains a frequent site of first progression, and treatment options after failure of both drug classes are limited [4, 17, 50]. A locally manufactured payload differs from intermittent systemic dosing by providing sustained exposure within the lesion. Its potential value therefore lies in the duration and spatial extent of local delivery rather than barrier circumvention alone.

Two potential applications follow. First, combination therapy may exploit complementary routes of exposure. Anti-HER2 antibody–secreting NSCs combined with tucatinib prolonged survival beyond either agent alone [19]. Coverage defines the spatial contribution of the cellular component and the regions that remain dependent on systemic treatment. Second, local delivery may be relevant when intracranial disease progresses despite extracranial control. In both settings, the operative question is not whether antibody can enter the central nervous system, but how much of a lesion is exposed and for how long. These possibilities remain translational hypotheses rather than conclusions of the present analysis.

Coverage provides information that survival alone cannot. The deposited antibody field was organized into domains approximately 100 μm across, a scale consistent with the modeled radius of secreted-antibody action. This concordance suggests that final coverage depends on where carrier cells localize and how their diffusion fields overlap. It does not, however, directly identify carrier-cell positions or exclude local differences in antibody production, tissue binding, clearance, or antigen availability. Direct colocalization of NSCs and antibody would be required to resolve these mechanisms.

This distinction suggests different optimization strategies. Broader carrier-cell distribution might be achieved through multisite or track-based injection, convection-enhanced delivery, or intraventricular administration [29, 45]. Laminin, fibronectin, or fibrin scaffolds may support cell survival and migration [51, 52]. Convection-enhanced delivery has defined determinants of intraparenchymal distribution and has enabled chronic intratumoral infusion in patients [53, 54]. Increasing antibody output per cell would address a different limitation by intensifying exposure where cells already localize. A brain-penetrant systemic agent may instead complement local delivery by covering tumor regions reached less efficiently by carrier cells [19]. These approaches require direct testing because the current data do not predict their incremental coverage.

The depth profile identifies the principal spatial deficit of the single-dose strategy. Coverage was greatest in the tumor core and declined toward both poles, with the steepest loss at depth. This pattern is not aligned with the clinical need to control residual disease at tumor margins after local therapy. It does not negate the substantial mean exposure achieved, but it identifies where altered carrier placement or additional dosing should be evaluated.

Several limitations define the interpretation of these findings. Direct intracranial implantation establishes an intracranial BT474Br xenograft but does not recapitulate the complete metastatic cascade. Differences in tumor architecture and microenvironment may influence NSC migration and antibody distribution; validation in spontaneous or experimentally disseminated models is therefore warranted. The reconstruction was obtained from one lesion sampled with consecutive sections spanning the entire tumor depth. It provides dense spatial information but no between-animal replication; the 60.6% value is therefore an observed lesion-level benchmark, not a population estimate, and the changepoints describe this lesion rather than a general tumor geometry. DAB-positive area indicates histologically detectable human IgG but does not establish antibody concentration, receptor occupancy, or functional exposure, nor can it distinguish extracellular, HER2-bound, and internalized antibody. NSC localization was not independently mapped, precluding direct colocalization of carrier cells with antibody deposition and limiting mechanistic attribution of the observed spatial pattern. Coverage was measured once, 48 h after administration, and cannot determine whether peripheral exposure later increases with migration or declines with carrier-cell clearance. The survival and coverage experiments used different treatment time points because the latter required a macroscopically established lesion for serial spatial reconstruction; accordingly, the day-7 survival benefit does not directly establish the therapeutic efficacy of the coverage field measured after treatment on day 20. Only one administration was evaluated by design to isolate the spatial field generated by a single dose. Although repeated dosing is feasible preclinically and clinically [24, 46], whether subsequent doses expand coverage into previously unexposed regions, prolong exposure, or reinforce the same field remains unknown. BT474Br was selected because few models combine native HER2 overexpression, brain tropism, and anti-HER2 responsiveness [55–57], but a single line does not represent the heterogeneity of clinical HER2-positive brain metastases. Finally, the immunodeficient host excludes adaptive immunity and antibody-dependent cellular cytotoxicity.

For two decades, cell-based delivery to the brain has been evaluated primarily by survival. That endpoint supported progression to first-in-human trials and randomized testing [25], but it does not indicate whether a platform is limited by payload activity or spatial reach. As locoregional strategies expand to CAR T cells, mesenchymal carriers, and intraventricular delivery [26–29], a common measure of delivered tumor fraction may enable more informative comparisons. Coverage converts an assumed property of a delivery platform into a quantity that can be measured and optimized.

## Conclusion

In a densely reconstructed intracranial BT474Br xenograft, a single dose of anti-HER2 antibody–secreting NSCs produced a mean section-level coverage of 60.6%, with spatially organized deposition that was greatest in the tumor core and diminished toward both poles. In a separate experiment, the same cell dose prolonged survival relative to cell-matched controls. These findings establish tumor coverage as a measurable preclinical endpoint and provide an initial lesion-level benchmark for optimizing cell-based intracranial delivery.

## Data Availability

The data generated in this study are available within the article. Analysis code used for the survival and spatial statistics is available upon reasonable request.
